# Becoming Culturally Competent: Transformative Learning in an International, Interprofessional Nursing Placement

**DOI:** 10.1177/23333936261450418

**Published:** 2026-05-15

**Authors:** Rachel A. Elliott, Heather Sweet, Natalie Bownes, Bayan Nasra, Kelsey Schumacher, Kelly-Anne Vanderdoe, Gabriela Digesu, Eunice Kungu, Grace Okello, Emma Crew, Mia Chauvin, Kendall Soucie

**Affiliations:** 1 University of Windsor, ON, Canada

**Keywords:** narrative inquiry, interprofessional education, international clinical placement, global health, nursing education, cultural competence, Tanzania

## Abstract

Research indicates that cultural competence is lacking among registered nurses, and nurses commonly report feeling ill-equipped to care for culturally diverse patients. This qualitative study employed a blended methodological approach informed by narrative inquiry and participatory action research to explore undergraduate nursing students’ development of cultural competence during an international, interprofessional clinical placement. Ten undergraduate nursing students completed a 3-week clinical placement in partnership with preservice teachers from the Faculty of Education. Aligned with participatory action research commitments to democratizing knowledge production, students were engaged as co-authors across multiple stages of the study. This approach recognized students as knowledge holders whose experiential insights shaped the analytic process. Data were generated through semi-structured interviews, and reflexive thematic analysis was undertaken iteratively, emphasizing the co-construction of themes. Five themes were generated: (1) opportunities for personal growth, (2) relationship building to facilitate collaboration, (3) interprofessional teamwork and leadership skills, (4) cultural competence development through immersion, and (5) shifting mindsets and learning from resource-limited settings. Findings underscore how structured reflection and debriefing can deepen equity-oriented learning by enabling students to critically examine assumptions, power, and privilege. These insights support the integration of international and interprofessional placements, alongside reflective pedagogy, into undergraduate nursing curricula.

## Introduction

Embedding cultural competence in undergraduate nursing education is essential for achieving equitable health outcomes (Jeffreys, 2016). Diversity in healthcare settings and the increasing need to engage with patients from diverse cultural backgrounds underscore the importance of preparing nurses to provide culturally competent nursing care. Cultural competence is the ability to deliver safe, high-quality, and effective care that respects patients’ cultural backgrounds and avoids disregarding or infringing upon culturally sensitive beliefs or practices (Antón-Solanas et al., 2021). However, research indicates that cultural competence is lacking among registered nurses (Andreassen et al., 2025; Shahzad et al., 2021), and nurses commonly report feeling ill-equipped to care for culturally diverse patients (Markey & Okantey, 2019; O’Brien et al., 2021). Despite the need to provide culturally competent care, it remains unclear which educational interventions or learning opportunities best prepare undergraduate nursing students and improve patient outcomes (Červený et al., 2022; Jongen et al., 2018; Osmancevic et al., 2025).

Over the past decade, the need to enhance the cultural competence of healthcare professionals has been widely recognized, as evidenced by the proliferation of educational interventions and training programs (Kaihlanen et al., 2019). Despite these efforts, the educational interventions and pedagogical approaches used to develop cultural competence among undergraduate nursing students remain poorly defined in the literature (O’Brien et al., 2021). International clinical placements (ICP) have been shown to provide rich experiential learning environments that expose students to diverse health systems and cultural contexts, offering opportunities to cultivate self-awareness and global nursing perspectives (Andreassen et al., 2025; Louis et al., 2025). However, little is known about how participation in international and interprofessional clinical experiences (IICP) influences the development of cultural competence among undergraduate nursing students. The existing literature examines these elements separately, leaving a gap in understanding how their intersection may contribute to transformative learning and culturally responsive practice (Andreassen et al., 2025; Louis et al., 2025; O’Brien et al., 2021). The purpose of this study was to (a) explore the experiences of Canadian undergraduate nursing students in an IICP and (b) to understand how this clinical experience influenced their development of cultural competence.

## Background

### Cultural Competence

Cultural competence is an ongoing process of developing the knowledge, skills, and attitudes necessary to provide effective healthcare for culturally diverse patients, considering their cultural behaviors, beliefs, and needs across diverse care settings (Campinha-Bacote, 2023; Garneau & Pepin, 2014; Papadopoulos, 2018). Curtis et al. (2025) extend the definition to ensure that the delivery of high-quality healthcare services contributes to achieving equitable health outcomes for all groups. Thus, the ability to practice culturally competent care is gained through continuous effort, practical education, and organizational support (Sharifi et al., 2019). Cultural competence is framed as an ongoing learning process rather than the achievement of full competence in cultures beyond one’s own (Curtis et al., 2025), requiring health professionals to engage in ongoing reflection and adaptation of their practice (Campinha-Bacote, 2023; Curtis et al., 2025; Papadopoulos, 2018). Cultural competence is integral to undergraduate nursing education (Canadian Association of Schools of Nursing [CASN], 2025).

Although undergraduate nursing programs integrate cultural competence into curricula to meet entry-to-practice competencies, there is limited evidence on the most effective approaches to doing so (Andreassen et al., 2025; Louis et al., 2025; Sjögren Forss et al., 2019). Although students report understanding concepts, many feel unable to apply this knowledge in clinical settings (Braithwaite et al., 2022). As cultural competence is an attainable skill that can be cultivated through education and training experiences (Stubbe, 2020), undergraduate programs should be encouraged to provide the necessary supports and practical tools to help students translate these skills into meaningful action (CASN, 2025).

### Theoretical Interventions to Develop Cultural Competency

There is limited research examining effective educational strategies to enhance cultural competence among undergraduate nursing students and within clinical practice settings (Červený et al., 2022). A systematic review by Oikarainen et al. (2019) identified that lectures, case studies, group discussions, and reflective activities improved nurses’ self-assessed cultural competence. Osmancevic et al. (2025) found that technology-based interventions, such as mobile applications and web-based training, enhanced cultural awareness, sensitivity, and communication skills. Similarly, Fadaeinia et al. (2022) evaluated an online cultural care training program among postgraduate nursing students. They reported higher post-intervention scores in cultural competence and self-efficacy, although these differences were not statistically significant. This finding is consistent with other scoping reviews of cultural competency training programs that found no difference in patient outcomes despite self-reported practitioner knowledge (Jongen et al., 2018). These findings suggest that integrating diverse, evidence-based or technological tools into nursing curricula may enhance learners’ understanding of how culture influences health and healthcare delivery. However, as Shopo et al. (2025) emphasize, cultural competence education is most effective when it is purposefully embedded across theoretical and clinical learning experiences.

A recent systematic review highlights the power of including storytelling and narratives to enhance learning about cultural competence (Osmancevic et al., 2025). Storytelling creates space for learners to engage with lived experience, positioning knowledge as relational and contextually situated rather than abstract or decontextualized (McAdams, 2001). In doing so, it supports meaning-making processes that are central to understanding how students interpret and internalize cultural encounters. Interactive strategies such as storytelling and group dialogue foster critical reflection and deepen empathy (Nashwan, 2024). Storytelling, for instance, allows students to consider multiple cultural perspectives. By hearing and reflecting on diverse narratives, students are better able to recognize how cultural beliefs, values, and healthcare practices are shaped by context, thereby challenging assumptions and broadening their understanding. However, without structured learning opportunities, students may struggle to reflect critically and provide culturally safe and equitable care (Majda et al., 2021; Tosun et al., 2021). Nurse educators have a responsibility to engage in critical self-reflection and apply evidence-based approaches to design inclusive learning experiences (Farokhzadian et al., 2022; Sjögren Forss et al., 2019).

### Experiential Educational Interventions for Developing Cultural Competency

Some nursing regulatory bodies in Canada have acknowledged that cultural competence should be an entry-to-practice requirement (College of Nurses of Ontario, 2019). Despite increasing educational initiatives to enhance cultural competence, nursing students lack practical experience and exposure to culturally diverse populations (Antón-Solanas et al., 2021; Tosun et al., 2021; Visiers-Jiménez et al., 2025). In some areas, limited exposure to diverse patient populations also restricts students’ readiness for culturally competent nursing practice (Antón-Solanas et al., 2021; Visiers-Jiménez et al., 2025). An integrative review by O’Brien et al. (2021) identified several effective strategies in undergraduate curricula, including simulation and patient scenarios incorporating cultural contexts, and cross-cultural clinical experiences. Simulation has been recognized as an innovative pedagogical strategy for developing cultural competence, delivered effectively through both face-to-face and virtual modalities (Byrne, 2020; Chung & Jarvill, 2019; Fung et al., 2023; Kessler & Kost, 2021; Qin & Chaimongkol, 2021; Shopo et al., 2025). Participation in such simulations has been shown to increase awareness of how cultural factors shape patient care and improve interprofessional communication (Červený et al., 2022; Costello et al., 2017). When purposefully integrated into nursing curricula, simulation-based education supports experiential learning and helps students translate theoretical knowledge into practice (Cant & Cooper, 2017). However, a systematic review of simulation-based interventions reported methodological limitations and inconsistent designs, limiting conclusions about effectiveness (Walshe et al., 2022).

The diverse use of standardized patient simulations has also improved students’ knowledge, skills, attitudes, and confidence in providing culturally congruent care (Ozkara San, 2019). Students valued the opportunity to practice cultural assessment in a supportive, risk-free environment and reported that these simulations helped them meet course objectives (Ozkara San, 2019). In fact, some student exchange programs or ICPs have drawn on research incorporating standardized-patient and simulation experiences as supplements to traditional teaching to develop cultural competence (Byrne, 2020) and to use culturally diverse students to promote collaborative learning (Shopo et al., 2025). Exposure to different cultures enhances students’ understanding of how cultural factors influence health and care delivery (Louis et al., 2025; O’Brien et al., 2021). Overall, combining experiential learning with structured debriefing and reflection has been shown to be effective in promoting culturally competent practice in undergraduate nursing education (James & Al-Kofahy, 2020).

### The Impact of International Clinical Placements

Service-learning and ICP are used in nursing education to promote cultural learning and equitable global care (Louis et al., 2025; Tinkler & Tinkler, 2016). Cultural immersion has been identified as a key pedagogical approach for developing cultural competence (Shopo et al., 2025). Exposure to unfamiliar health systems and sociocultural contexts through immersion enables students to broaden their global perspectives, strengthen clinical reasoning, and build self-efficacy (Browne et al., 2015; Louis et al., 2025). Empirical evidence further demonstrates that ICP are associated with measurable improvements in cultural competence, particularly when students engage with diverse and resource-limited communities (Andreassen et al., 2025; Bagnasco et al., 2020; Gower et al., 2023).

The effectiveness of ICP is enhanced when placements are intentionally structured and supported by pre-departure preparation and guided reflection. Ulvund and Mordal (2017) found that even short-term ICP can meaningfully strengthen cultural competence when reflective learning processes are embedded before, during, and after the experience. Through these reflective practices, students move beyond stereotypes, develop cultural humility, and deepen empathy and open-mindedness (Gower et al., 2023; Hovland & Johannessen, 2018). Although such experiences may vary across contexts, ICP heighten awareness of social determinants of health, including poverty, inequity, and health system constraints, often prompting increased advocacy for marginalized populations (Gower et al., 2023).

ICP are consistently described by students as transformative, shaping both personal and professional identity development (Ulvund et al., 2023). Participants frequently characterize these placements as “eye-opening” or pivotal learning moments and report increased confidence, adaptability, and capacity to manage emotionally demanding situations in practice (Johannessen et al., 2014; Kohlbry, 2016; Murray, 2015; Reid-Searl et al., 2011; Tuckett & Crompton, 2013). Graduates often advocate for the integration of ICP as a required component of nursing curricula, citing its role in enhancing cultural responsiveness, improving nursing practice, and strengthening understanding of global healthcare systems (Gower et al., 2023; Kohlbry, 2016; Ulvund et al., 2023).

### The Value of Interprofessional Education

Interprofessional education (IPE) is widely recognized as foundational for the training of healthcare professionals, as it strengthens collaborative practice, interprofessional communication, and teamwork among learners from multiple health disciplines (Patel et al., 2025). According to the World Health Organization (2010), IPE involves students from multiple professions learning about, from, and with one another to cultivate skills essential to interprofessional collaborative practice. A crucial element of IPE is students’ enhanced understanding of the roles and responsibilities of their own and other professions (Patel et al., 2025). Early exposure to IPE in undergraduate education helps nursing students recognize the diverse roles, cultures, and expertise of team members and promotes effective and respectful communication across professional and cultural boundaries (Zenani et al., 2023). Simulation-based IPE has also demonstrated an ability to enhance collaboration and improve outcomes (Banks et al., 2019). IPE can help learners achieve or improve interprofessional communication and facilitate effective teamwork (Friedrich et al., 2021). Evidence consistently indicates that strong interprofessional collaboration enhances care delivery and improves patient safety outcomes (Homeyer et al., 2018; Reeves et al., 2016).

### Interprofessional and International Clinical Placements

While the value of ICP has been documented (Andreassen et al., 2025; Bagnasco et al., 2020; Louis et al., 2025), very few studies have explored how participation in IICP influences undergraduate nursing students’ cultural competence and collaborative practice development. Much of the existing research focuses on isolated professional experiences, with fewer studies explicitly capturing interprofessional learning processes, impacts, or outcomes within international clinical contexts. For example, McElfish et al. (2018) integrated interprofessional education and cultural competency training for interdisciplinary student teams, including medical, nursing, and pharmacy students; this program produced statistically significant improvements in participants’ self-reported cultural competence and readiness for collaborative practice across professions. However, these positive findings are largely drawn from domestic settings and do not account for the added complexity of cultural immersion within low-resource, global health contexts (McElfish et al., 2018). As a result, how nursing students learn *with, from, and about* other disciplines during international placements remains largely underexplored. Consequently, there remains a critical gap in understanding how interprofessional learning unfolds during international clinical experiences and how such placements contribute to the development of cultural competence skills among undergraduate nursing students. This gap underscores the need for qualitative inquiry that centers students’ lived experiences within IICP and captures the mechanisms through which cultural learning and collaborative practice are fostered, particularly through storytelling. Thus, the purpose of this study was to explore, through a blend of narrative inquiry and participatory approaches, the development of cultural competence among Canadian undergraduate nursing students during an IICP. We also sought to understand how this clinical experience impacted their development of cultural competence. The following research questions guided this inquiry:

1. How do third-year undergraduate nursing students make meaning of their interprofessional and international clinical experiences in relation to culture, identity, and nursing practice?
2. What insights do third-year undergraduate nursing students construct from interprofessional and international clinical experiences?
3. How do these meanings/learnings inform their understanding of culture and care?

### Theoretical Framework

Campinha-Bacote’s (2002, 2018) model of cultural competence in healthcare offers a comprehensive framework for delivering effective care in diverse cultural contexts. Campinha-Bacote (2002) defines cultural competence as the convergence of behaviors, attitudes, and policies embedded within systems, agencies, or professional practice that enables individuals and organizations to operate effectively in cross-cultural encounters. Cultural encounters and lived experiences may facilitate the development of healthcare professionals’ knowledge and help translate this knowledge into practice within a cultural setting (Byrne, 2020).

Campinha-Bacote (2023) argues that the development of cultural competence begins with cultural encounters and experiences. Rather than a static goal, cultural competence is a continuous journey rooted in self-reflection, meaning-making, and lifelong learning. Cultural encounters represent the practical dimension of the concept, in which nurses provide care to culturally diverse people and support their use of affective and cognitive skills in practice (Bagnasco et al., 2020; Campinha-Bacote, 2002, 2023). Cultural competency cannot occur without exposure to diversity, even when cultural desire and awareness are present (Campinha-Bacote, 2023).

Central to this model is the concept of “*cultural competemility*,” a term coined to describe the integration of cultural humility and cultural competence. Cultural competemility reflects a dynamic and evolving process in which cultural humility informs and permeates the five core components of cultural competence: awareness, knowledge, skill, encounters, and desire (Table 1). Within the context of the IICP, Campinha-Bacote’s model provides a useful lens for understanding how students’ learning unfolded through immersion and practice, particularly through sustained cultural encounters with Tanzanian communities and interprofessional partners, which could serve as catalysts for cultural competence.

**Table 1.** The Five Constructs of Cultural Competence.

Construct	Definition
Cultural Awareness	The deliberate self-examination of one’s own cultural identity, beliefs, values, and biases, and how these may influence clinical interactions and care.
Cultural Knowledge	The process of acquiring information about different cultural groups, including their worldviews, health-related beliefs, and practices.
Cultural Skill	The ability to effectively gather cultural data and perform culturally appropriate health assessments and interventions.
Cultural Encounters	Direct engagement with individuals from diverse backgrounds enables the refinement of one’s understanding and the reduction of stereotyping.
Cultural Desire	The internal motivation to genuinely engage in the lifelong process of becoming culturally competent, driven by a commitment to equitable care.

*Source*. Adapted from Campinha-Bacote (2018).

*Note.* The process of cultural competence in the delivery of healthcare services: A model of care.

## Methods

This study employed a blended methodological approach informed by narrative inquiry (NI) and participatory action research (PAR) to explore undergraduate nursing students’ experiences of culturally competent care during an international, interprofessional clinical placement in Tanzania. NI is grounded in the belief that individuals make sense of their lives and experiences through storytelling (Connelly & Clandinin, 1999). This approach enabled an in-depth exploration of students’ evolving personal and professional identities by attending to temporality (past, present, and future), place (Tanzania as a site of learning), and sociality (relational and emotional contexts), thereby providing a rich understanding of how meaning was constructed through experience.

In alignment with PAR principles, students were engaged as co-researchers and co-authors throughout the research process, including study design, data analysis, and knowledge translation (Cruz et al., 2017; Kemmis et al., 2014). PAR supports collaboration and reflexivity while challenging traditional power hierarchies in knowledge production (Higginbottom & Liamputtong, 2017). Integrating NI with PAR enabled the co-creation of knowledge that was not only methodologically rigorous but also relational, contextually grounded, and responsive to students’ lived experiences within an international and interprofessional learning environment.

## Study Setting and Recruitment

### Setting

This 3-week IICP was implemented in two regions of Tanzania. Tanzania is a lower-middle-income country located in East Africa, with a population of approximately 69 million people (World Bank, 2024). It is culturally and geographically diverse. The first week took place in Moshi, a city in the Kilimanjaro Region of northeastern Tanzania, home to several ethnic groups, including the Maasai. Swahili is the primary language spoken, while English is commonly used in tourism and among individuals with secondary or higher education. The second half of the clinical placement was conducted in Singida, a predominantly rural region in central Tanzania, where agriculture remains the primary source of livelihood for most residents (Bundala et al., 2025).

### Clinical Experience

Before acceptance to the IICP in Tanzania, students were committed to participating in weekly interprofessional preparatory meetings for the winter semester (January–April). In addition to their third-year courses, students were permitted to enroll in a fourth-year Global and Planetary Health core nursing course to serve as an educational foundation for the health-teaching topics they selected. Students used their coursework and pre-departure meetings to prepare health promotion and educational activities on topics such as menstruation, gender-based violence, HIV/AIDS, and oral hygiene, which were delivered by IICP students during the Girls Leadership and Empowerment Program (GLEP), a week-long girls’ empowerment initiative for 60 local girls and their teachers. In Moshi, students toured local dispensaries and a district hospital led by local healthcare providers and organized by a Tanzanian physician practicing in the community. The team led a screening clinic for non-communicable diseases under the direction of a local Maasai physician with the goal of increasing positive interactions with healthcare workers and bringing attention to non-communicable diseases.

In Singida, the group led and participated in a community outreach screening clinic for non-communicable diseases, including hypertension and high blood glucose, in collaboration with the healthcare team from a local dispensary. The students also participated in a community-based albinism outreach initiative, providing protective resources such as sunscreen and hats to support skin health and health teaching to prevent complications. Students also shadowed the healthcare team of nurses and physicians at district health hospitals and local dispensary clinics in specialty areas, including maternal-newborn immunization and child-health clinics, triage, and wound care wards, to gain insight into the interprofessional practice in resource-limited settings.

### Participants

The single criterion for study inclusion was enrollment in the third-year interprofessional clinical consolidation experience in Tanzania. All students (*n* = 10) consented to participate and self-identified as female, ranging in age from 21 to 38. One student identifies as African Canadian, one as African, one as Indigenous (Métis), one as Middle Eastern Canadian, and the remaining six as Caucasian. Six participants were born in Canada, and four were born outside Canada, including one refugee who immigrated to Canada as a child. Four participants were second-career learners, while the remaining six students were direct entrants to the undergraduate nursing program at a southwestern Ontario university.

### Recruitment

The study involved 10 third-year undergraduate nursing students at a Canadian university. These students applied and were selected to participate in a 3-week clinical consolidation placement in Tanzania, focused on health promotion and community-based nursing care, in collaboration with the Teach Tanzania program, which is run through the university’s Faculty of Education. Upon completion of the IICP, all participating nursing students were engaged as co-authors and invited to participate via email by our clinical lead (NB).

## Data Collection

In alignment with participatory principles, students (participants) could also select their preferred interviewer (RE, HE, or NB) and indicate whether they would like peer support, with a student interviewer present. In this case, the interview was conducted in a dyadic format, with the faculty and student interviewers present. Interviews were conducted as conversational, participant-led accounts to support an unfolding, flexible narrative and to foreground lived experience across time and context (McAdams, 2001). This approach aligns with narrative inquiry, attending to how participants storied their experiences, made meaning of significant moments, and reflected on how these experiences shaped their evolving identities as nurses. Participation or non-participation had no impact on students’ academic standing. Participants were offered a $25 gift card as an honorarium.

Two sources of narrative data were collected:

1. *Semi-structured interviews* (*n* = 10) were conducted 2 to 3 weeks after the placement was finished. The semi-structured interview guide was co-created with students and designed to invite storytelling rather than discrete responses (Supplemental Material). Interview prompts encouraged students to describe their experiences, expectations, critical learning moments, cultural encounters, and emotional responses, and to reflect on how these moments unfolded over time and impacted their nursing practice. Participants were invited to consider how they made sense of these experiences, how their perspectives shifted, and what these experiences revealed about their emerging professional and personal identities as nurses. Additional prompts explored expectations, critical learning moments, cultural encounters, emotional responses, and perceived impacts on nursing practice. Interviews ranged from 40 to 70 min, were audio recorded on Microsoft Teams®, transcribed verbatim, and anonymized by the student interviewed. Students were also invited to review their transcripts as narrative accounts and were permitted to redact any portions of their stories they did not wish to share with the research team prior to data analysis.
2. Reflective journals were written from the students’ perspective during or immediately after the placement, documenting students’ insights and evolving understanding of their professional roles within immersive cultural experiences. Two formal reflective journals were required for this clinical experience, but not for data collection. Students could choose to include their reflective journals in this study. All 10 students who participated chose to include their two formal reflective journals. These journals were approached as narrative artifacts that captured participants’ ongoing processes of sense-making, including revisiting experiences, interpreting their significance, and tracing shifts in understanding over time. Although students engaged in near-daily informal journaling while in Tanzania, they chose not to include these entries; instead, they drew on the stories within them during analysis to “burrow deeper” into meaning and to extend reflection (Connelly & Clandinin, 1999).

## Data Analysis Strategy

Following the placement, these two data sources were triangulated to develop a comprehensive understanding of student learning during and after the placement and to assess the impact on the development of cultural competence. Consistent with narrative inquiry, both data sources were understood as complementary narrative data sources through which participants constructed meaning from their experiences across time (Connelly & Clandinin, 1999; Page et al., 2013). Reflective journals documented in-the-moment meaning-making during or immediately following the IICP, whereas interviews supported retrospective reflection and interpretation after students returned home.

All interview transcripts and reflective journals were analyzed together as a single analytic dataset using reflexive thematic analysis (RTA; Braun & Clarke, 2022), with an inductive approach that treated each participant’s interview and corresponding journals as a linked narrative set. The same coding framework was applied across both data sources, attending to semantic and latent meanings, as well as to how experiences were storied and reinterpreted over time. Reflective journals often informed early experiential and emotional codes (e.g., cultural shock, vulnerability, and relational learning), while interview narratives elaborated, reinterpreted, or consolidated these reflections. Differences between contemporaneous journal entries and retrospective interview accounts were treated as analytically meaningful shifts in narrative understanding rather than inconsistencies.

Students who chose to participate in data analysis (*n* = 8) were given three transcripts to review. Initial coding was open and unstructured, and students were encouraged to add memos and insights throughout their engagement with the data. Two faculty members (NB and HS) were assigned half of the transcripts for coding, while the faculty adviser and primary researcher (RE) coded all 10 transcripts. Students and faculty participated in three formal collaborative analytic sessions, during which they reviewed narrative data, engaged in dialogic meaning-making, and contributed to the identification, refinement, and co-construction of preliminary themes (Higginbottom & Liamputtong, 2017).

Students then applied the co-developed coding framework more systematically to a new set of transcripts. Throughout the analytic process, reflexive journaling and regular team meetings and discussions were used to enhance rigour, interrogate assumptions, and attend to power and positionality. Through iterative team discussions, themes were identified and refined, after which students reviewed additional transcripts and selected a theme to further develop. Students met with the faculty adviser (RE) when they needed additional guidance to facilitate the writing process for their selected theme. Working in pairs, students deepened their analysis within their chosen theme, prepared a draft interpretation, and presented it to faculty and peers at the final data analysis team meeting for collective reflection and refinement. Themes were ultimately developed collaboratively through ongoing iteration across both data sources, supported by reflexive memo-writing, team discussions, and attention to the researchers’ positionality. Integrating interviews and reflective journals in this way enhanced analytical depth and supported a comprehensive understanding of students’ development of cultural competence within an IICP.

## Researcher Positionality

The faculty team comprised educators in clinical instruction (RE, HS), program leadership (NB), and qualitative research mentorship (KS), while student co-authors were both participants and emerging researchers. This dual positioning reflects the study’s participatory orientation, in which students were engaged as knowledge contributors and collaborators in interpretation rather than solely as subjects of inquiry.

Student participants brought diverse lived experiences that shaped both the data and its interpretation. All participants identified as female and represented a range of cultural, racial, and experiential backgrounds, including African Canadian, African, Indigenous (Métis), Middle Eastern Canadian, and Caucasian identities. Participants also varied in age, migration histories, and educational trajectories, including second-career learners and those entering directly into undergraduate nursing education. These intersecting identities and experiences informed how students engaged with the IICP, the stories they shared, and the meanings they constructed in relation to culture, power, and professional identity. Student co-authors drew on these lived experiences in analysis, contributing insider perspectives that supported more nuanced and contextually grounded interpretations of narrative accounts.

In contrast, the faculty team identified as white women and occupied positions of institutional authority within a Canadian academic context, with varying degrees of social and professional privilege. The team recognizes that these positionalities are embedded within broader historical and ongoing colonial structures that shape global health education, including international clinical placements in low- and middle-income countries. These dynamics carry the risk of reinforcing inequitable power relations, privileging Western knowledge systems, and reproducing deficit-oriented understandings of host communities. As such, the research team remained attentive to how their social locations and institutional roles may have influenced how stories were elicited, interpreted, and represented, as well as whose perspectives were centered or marginalized.

Consistent with narrative inquiry, analysis was understood as a relational and interpretive process involving the co-construction and re-storying of participants’ experiences rather than their objective representation. Ongoing reflexive practices were used to interrogate how assumptions, disciplinary training, and cultural positioning shaped analytic decisions. Attention was directed toward disrupting deficit-based narratives, attending to relationality and reciprocity, and foregrounding strengths-based, contextually grounded understandings of practice. Through this reflexive and collaborative approach, positionality was treated as integral to the analytic process. The team sought to engage critically with the ethical and epistemological tensions inherent in studying international clinical experiences, while remaining accountable to participants’ stories and to broader commitments to equity and social justice.

## Ethical Considerations

We acknowledge the generous funding from the University Diversity, Indigeneity, and Anti-racism Professional Development grant that supported this research. This study was approved by the University of Windsor’s Research Ethics Board [#25-062]. Students were involved in the ethics submission and co-creation of all aspects of the study design. After completing the clinical consolidation course and submitting their final grades, students were invited to participate and collaborate on this project as co-authors. Written informed consent was obtained prior to student participation in the semi-structured interview. All study-related data were deidentified by RE and stored on a password-protected computer.

## Findings

Consistent with NI, we understand knowledge as relationally and contextually co-constructed, shaped through interactions among researchers, participants, and the Tanzanian clinical environment. The findings represent interpretive threads constructed from students’ narratives rather than categorical themes, illuminating how students made sense of their learning about cultural competency across time, place, and relationships. Participants’ narratives demonstrate how meaning was reconstructed over time, with earlier moments of discomfort later re-storied as critical learning experiences. This temporal and relational re-authoring of experience reflects core narrative inquiry commitments to understanding how individuals make sense of practice through lived and remembered stories.

Five major themes were constructed from the overall analysis: (1) Opportunities for Personal Growth, (2) Relationship Building to Facilitate Collaboration, (3) Interprofessional Teamwork and Leadership Skills, (4) Developing Cultural Competence through Immersion, and (5) Shifting Mindsets and Learning from Resource-limited Settings.

### Opportunities for Personal Growth

Early in the IICP, participants expressed uncertainty about how to position themselves within the clinical environment. Over time, these same situations were retold as formative experiences that reshaped their understanding of both personal and professional identity. Ultimately, students shared that the IICP fostered a sense of purpose, enhanced self-confidence and self-awareness, and contributed to personal and professional growth. Informal reflective journaling on their personal and clinical experiences, and debriefing sessions as a clinical group, helped students identify their strengths and opportunities for growth, ultimately enhancing high-level professional skills. Reflection provided students with an opportunity to demonstrate humility and highlight successful interactions in a new cultural context. When retelling their story, one student acknowledged their daily journaling was an enjoyable experience, stating, “*I think that [writing in my journal] kind of showed me how important reflecting is and how beneficial it can be for your self-growth and just being a better nurse in general*” (Participant 1). Structured debriefings and daily reflection opportunities enabled students to engage in meaning-making and to reflect on their personal values, beliefs, and cultural worldviews. This helped students enter clinical encounters with an open attitude and respect for cultural differences. Students learned to expect the unexpected, which was described by one student who explained how this IICP “*encouraged students to go beyond their comfort zones and become comfortable with the uncomfortable and not only to do that, but to also explore their own identities and their own skills*” (Participant 5). Students’ stories frequently described moments of dislocation, where familiar clinical routines and cultural expectations no longer provided a reliable guide for action. Students felt that practicing “*outside their comfort zone*” created space for them to develop understanding within the cross-cultural learning environment.

The IICP provided opportunities for students to enhance their self-confidence by providing unique clinical experiences. Immersion helped students to be flexible and adapt, which was shown when a student had to lead a community clinic as a “charge nurse.” The student recalled, “*at first, I felt really overwhelmed . . . but being pushed to adapt and take charge in that environment made it even more powerful and meaningful. It helped build my confidence*” (Participant 9). Many students highlighted that they are rarely provided with leadership opportunities in their clinical rotations within the Canadian healthcare system. In this IICP, students emphasized stories and shared experiences about building meaningful connections with the community and collaborating with local healthcare professionals. After a local physician partner emphasized the importance of building trust during community screening, it resonated with one student who shared, “*that shifted my perspective, as I realized it wasn’t about the numbers, it was about connection*” (Participant 10). Students also began to shift their mindset toward prioritizing connection with the community rather than task completion.

External opportunities to build collaborative partnerships significantly enhanced training prior to departure. Students worked to build relationships with a nonprofit organization that prepares and distributes sustainable menstrual solutions for girls and provides education on menstruation.

Before departure, I connected with a local Days for Girls chapter leader. Through coordinated fundraising and donations, we purchased 150 reusable menstrual pad kits, locally produced within Tanzania, to distribute during the GLEP program. This initiative represented a sustainable approach to menstrual health by providing essential supplies to girls, while simultaneously investing in a Tanzanian woman’s micro-enterprise to promote economic empowerment. This was one of the most impactful contributions I made to the IICP experience. (Reflective journal)

Ultimately, this learning allowed students to zoom out and focus on larger healthcare issues, where students adapted their engagement strategies to meet the community’s identified needs. As a result of these experiences, students were able to recognize their biases, assumptions, and privileges that may impact cultural encounters through continuous self and group reflection. One student stated, “*I realized how important it is not to make assumptions about a person’s background or beliefs. Even though I didn’t fully understand their culture. . .I made a conscious effort to ask questions and to approach every interaction with respect and openness*” (Participant 4). This starkly contrasts with a Canadian hospital clinical shift, where students often focus on task completion for one identified patient without considering culture. Ultimately, these leadership experiences challenged students to think beyond the clinical setting toward a sense of purpose and personal identity development. After engaging in group reflection, one student beautifully described how this lived experience shaped their thinking by reflecting: “*This experience has shaped not only the nurse I aspire to become but also the person I strive to be. . .*” (Reflective journal).

### Relationship Building to Facilitate Collaboration

Students built relationships that enabled them to work together to support one another and meet the competing demands of the IICP. One nursing student discussed how developing trusting relationships was critical to success: “*I loved how we all had to lean on each other. We didn’t know what we were walking into each day, but we worked together and made it happen*” (Participant 1). Working closely with education students created a unique opportunity for interprofessional collaboration across fields that would not typically intersect in traditional nursing education. Students emphasized that “*knowing one another*” was critical to strengthening relationships when implementing tasks such as health teaching presentations at the GLEP. A student explained, “*Coming together from two professions with the shared goal of empowering the girls created so much respect between us. We each brought something unique*” (Participant 1). Students highlighted from their reflective journals that building mutual trust between nursing and education students was essential for effective collaboration to enhance cultural competence.

Prior to the IICP, education and nursing students met weekly for 2 hr from January through the end of April and the beginning of the IICP. Although these training sessions were perceived as helpful, a significant gap was that this training and preparation occurred in silos, with the two groups not effectively merging until the first few days of the IICP in Tanzania. Despite initial challenges in integrating the two student groups, students described how, over time, their mutual reliance strengthened teamwork as they prepared, designed, and delivered their lessons in the GLEP. The overlap of shared skills and knowledge provided support when nursing students struggled in situations outside their professional domain, such as visiting elementary schools and assisting in classrooms. One student explained, “*going into the schools, you could tell the nurses were kind of closed off, and the teachers were in their element. But then, when they were presenting or doing their workshops, they [nursing students] just started to take action to help*” (Participant 5). Nursing students recognized, “*how much these roles mend together, and how you’re also teaching nursing topics and how you can learn from teachers to see just how they communicate with their students*” (Participant 5). One student reflected on the uniqueness of this IICP in comparison to a usual practicum: “*In Tanzania, we really had to rely on one another. At the end of the day, we needed each other and were always there for one another. By the end of it, we were like family*” (Participant 4). One student reflected how this vulnerability contributed to team growth, “*I think everyone within our team has something good to offer when someone was in need of support. Everyone had a different perspective to give or talk to you about. We were really, really good at that*” (Participant 8).

Students described how working alongside Tanzanian healthcare providers shifted their sense of professional identity and challenged traditional hierarchies. Despite identified language barriers, communication was effective between healthcare workers due to their ability to speak English. One student noted, *“I felt like my voice mattered in the clinic, which is different from Canada, where students often feel overlooked”* (Participant 9). Participant 1 emphasized, *“the doctor didn’t lecture at us, he spoke with us. He wanted to share his knowledge and hear our perspective, too.”* Similarly, another explained, *“There was a lot of mutual respect. Even though we were students, the local nurses and doctors treated us like part of the team”* (Participant 6). Ultimately, the mutual trust created space and allowed students to feel confident in their abilities as healthcare professionals. One student summarized this feeling: *“Working with the kids, the women, the local team—it just reminded me that healthcare is about human connection first”* (Participant 7). These connections enhanced understanding of what health means to the community and health care team members, and one student further shared: *“I realized healthcare isn’t just about skills or tasks, it’s about trust and relationships. That was transformational for me”* (Participant 10). Working with the local healthcare professionals was described as transformative, reshaping their view of nursing practice and relational approaches to interprofessional teamwork.

### Interprofessional Teamwork and Leadership Skills

The connection and open communication between team members and their ability to consistently engage in reflective praxis created opportunities for students to step into both leadership and followership roles. Every nursing student described at least one story where they were asked to challenge themselves by being a leader that would not necessarily arise in a regular clinical day. For example, one nursing student stated, “*I stepped into a leadership role during the school visit; we had to organize flow, communicate as a team, and make decisions quickly. I’d never done that before*” (Participant 3). Students attributed a significant reason for this success to the support system created within the team, which contributed significantly to self-confidence and professional growth. The student elaborated, “*I felt supported by my professors, as they helped me to take on my own challenges. I felt mentored and included, which helped with my confidence. To be included, to feel seen and be heard was something of great value*” (Participant 3).

Bonds between nursing and education students, instructors and the local healthcare team helped students to feel safe to step into direct leadership roles. For example, students were asked by a local physician to organize a community screening clinic in Moshi for noncommunicable diseases. Despite preparation and pre-briefing, nursing students arrived with supplies that needed organizing and without a coordinated implementation plan. One student stated: “*We discussed how the clinic might run, but we didn’t know exactly who we would be serving until we got on site*” (Participant 9). Students had to step into leadership roles to assess, respond, and adapt their communication and care-delivery methods, allowing them to communicate despite a language barrier in real time and modify their strategies. As nursing and education students worked together, many asked for screening in pairs or groups while asking health-related questions. While debriefing this experience, despite acknowledging strengths, students strategized to create a plan for the next clinic, including solutions like “*specific people documenting values, organizing individuals into a line rather than a crowd, and prioritizing those with specific conditions to be seen first*” (Reflective journal). Through debriefing, students critically reflected on their strengths and weaknesses, fostering teamwork and leadership while initiating constructive changes for the next clinic in Singida.

Students felt that this IICP enhanced their knowledge as students, allowing them to grasp more complex and abstract concepts, leading to the development of more well-rounded nurses as they enter their careers, compared to traditional clinical experiences. One student identified and discussed a gender-based critique of the GLEP in real time with the clinical instructor, observing that male community partners were leading the program’s initial introductions and discussions. The student illustrated, “*girls knew they would be attending a program about women’s leadership but were met with men in leadership roles. It should have been us, the women, standing in front of them, role-modelling what women in leadership roles can look like*” (Reflective journal). The student further analyzed this, which fostered a chance to challenge her thinking while being mindful of cultural values. The student recognized that to enhance the development of cultural competency, she “*had to adapt my thinking to encompass their traditional customs while brainstorming how we could do women’s empowerment justice*” (Participant 8). Furthermore, students related to the girls in the GLEP program, which facilitated a broader understanding of different cultural perspectives on various issues, like empowerment. One student reflected, “*I had one-on-one moments with them that truly touched my heart. The [girls] told me things like; you’re my sister and my role model. . .seeing someone who looks like them makes a huge difference! It’s such an important connection*” (Participant 4). This illustrates how students were encouraged to step into leadership roles and broaden their perspective beyond the immediate clinical shift, focusing on holistic learning rather than simply completing isolated skills or tasks.

### Developing Cultural Competence Through Immersion

Many students emphasized that they went into this IICP with little to no expectations, allowing them to remain open to cultural and professional differences in practice. Despite the preparation prior to departure, one student described “*walking into the unknown*” (Participant 8) and being surprised at the depth of learning compared to traditional classroom teaching strategies. Immersion was vital in helping students confront daily cultural experiences that challenged their previous assumptions and beliefs and re-negotiate taken for granted assumptions in clinical practice. Before departing, students developed their awareness of Tanzanian culture through intentional learning during weekly meetings and training sessions. A student recounted: “. . .the *Days for Girls Training that we did was very helpful*” (Participant 6). Most students agreed that advanced enrollment in the Global and Planetary Health course and weekly interprofessional team training enhanced their ability to build relationships and connect meaningfully. Cultural awareness was key to effective health teaching during the GLEP, ensuring a respectful approach to topics such as menstruation. Rather than delivering standardized Western health messaging, students first sought to understand local beliefs, language stigma, and gender norms surrounding menstruation. This required listening, adapting their teaching approaches, and critically reflecting on their own assumptions. By prioritizing dialogue over didactic instruction, using culturally appropriate language, and collaborating with local partners, they practiced cultural humility in action. In doing so, menstruation education shifted from simply transferring knowledge to co-creating understanding within the community context which is an essential component of cultural competence. Additionally, “unlearning” was something that students described as an essential part of the experience. Participants’ stories revealed how emotional responses, relational dynamics, and identity tensions shaped their learning. One student reflected on how critical cultural immersion was in laying the foundation for cultural competency:

To unlearn biases and go in with a clear slate and just learn from the people. . .to develop cultural competence, you need to experience [culture], you can’t just read about it. It’s one thing to watch a show on it or read a book, but it’s another thing to live it. (Participant 3)

Despite prior preparation and training, students were sometimes overwhelmed by cultural encounters. Students were welcomed with song and dance from the Maasai women when arriving to conduct their health screening clinic in Moshi. During the implementation of the clinic, the Maasai people were initially nervous to participate. One student reflected, “*Through discussion with the local physician we learned their initial apprehensiveness was due to their fear of receiving ‘bad news’ and the lack of financial aid available for follow up care should they receive abnormal results*” (Reflective journal). The local Maasai physician allowed students to perform screening on him as a way of building trust with the community. Subsequently, the Maasai people approached students to receive health screening. “*Large crowds gathered around us as the community found comfort in approaching with their family and friends at their side. . .it was overwhelming trying to conduct screening and translation for so many people at once, as many people were anxious to hear their results*” (Reflective journal). Through debriefing with staff, students were able to develop a plan for the next screening clinic that would allow for a more organized approach while establishing trust with the community. Concurrent debriefing was essential during this IICP. Students illustrated a deeper, more meaningful understanding of culturally safe practices through critical reflection, both informally and in formal, structured sessions. One student described a new appreciation of what culture and care mean to them upon witnessing, “*how these doctors and nurses were caring for their patients. . .truly appreciating that this is their culture and it’s what makes them feel safe*” (Participant 5). Students developed nuanced perspectives on varied health practices, religious beliefs, and community perceptions through direct engagement in the local clinical setting. Three students experienced a profound moment of human connection. They were able to: “*observe a C-section where the team began with a prayer and they blessed the mother and the child before they started any incision in the operating room. . .being included in such a sacred moment was truly an honour*” (Participant 5).

Local healthcare providers’ mentorship opportunities in the Singida wound care clinic helped a student “*understand how to provide competent care with limited resources*” (Participant 8). Students were challenged in real time to overcome cultural barriers to care, including language barriers. Immersion in this cross-cultural setting challenged students to use non-verbal communication skills. Students recounted that the “*language barrier forced you to consider different beliefs and healthcare systems and adapt your approach*” (Participant 2). Experiences of discomfort, uncertainty, and vulnerability were not simply reactions to a new environment but became central narrative turning points through which students reinterpreted their professional identities. Observing shared non-verbal communication practices, such as open body language, singing, dancing, and praying together, helped to cultivate a “brave space” that was culturally responsive and psychologically safe. This environment enabled both students and community members to examine and reshape their understanding of the therapeutic relationship. For example, during debriefing following a screening clinic for non-communicable diseases in Moshi, students questioned assumptions that participation in screening clinics is inherently safe or routine, recognizing that such expectations are shaped by Western healthcare norms. Through reflection, they began to consider the social, cultural, and historical contexts, including legacies of colonialism and inequitable health systems, that contribute to community members’ hesitancy to engage in screening. For example, *oloirobi* is a Maasai term used to describe health concerns and often serves as a broad, catch-all expression for illness. Through dialogue with the local healthcare team, students came to understand how this framing of illness may not align with biomedical diagnostic categories, potentially complicating the identification of non-communicable diseases and influencing pathways to care. By actively observing and modifying attitudes, students were able “*to comfort [patients]and tailor to their needs. This experience really switched my mindset from treat others how you want to be treated to treat others the way they want to be treated*” (Participant 8). Immersive consolidation experiences provide strong foundational knowledge to develop cultural competence in nursing students for use now and in their future careers.

It’s not just about being aware that you’re in a different culture; it’s about being respectful, open, and willing to adapt, even in small ways. You might not fully understand everything or be able to put yourself completely in someone else’s shoes but being mindful and open to learning from the community you’re in is so important. Even small things, like how you greet people or how you carry yourself in a new environment, can show a lot of respect. You’re going there not just to help, but to learn, and sometimes the most powerful part of the experience comes from letting go of assumptions and just listening. (Participant 9)

### Shifting Mindsets and Learning From Resource-Limited Settings

Students’ narratives positioned the IICP in Tanzania not simply as the location of a clinical practicum, but as a disruptive, meaning-generating place that unsettled familiar assumptions about health care. This unfamiliar clinical environment functioned as a catalyst and challenged students to reconsider what equity in practice truly means. One student reflected, “Seeing *nurses work directly with families in the community made me realize how much primary prevention matters. It’s not just about treating disease; it’s about educating and empowering people where they live*” (Participant 7). Limited resources created opportunities to demonstrate ingenuity and adaptive problem-solving. In the wound care clinic, one student described applying honey to the wound bed “*using a gloved hand to scoop it out of a sterilized, recycled food jar*” (Participant 3). One student reflected on an observational experience of a wound debridement in the clinic, “*the wound care nurse used debridement tools that had been thoughtfully sterilized in large plastic buckets. . .it was an opportunity to reflect on the waste in our own healthcare system*” (Reflective journal). Another recalled observing a cesarean section in which obstetric surgeons utilized a “*glass bottle to store iodine for disinfection*” (Reflective journal). These examples illustrate how evidence-informed interventions were thoughtfully adapted to the local context using available materials, while maintaining attention to sterility and patient safety. When reflecting on the experience of shadowing local nurses, one student shared, “*they provided the best quality care with so little. . .the patient wouldn’t even know they were lacking supplies*” (Participant 8). Similarly, Participant 5 emphasized, “*seeing how innovative they are with what they do have, and how they give the same care that children, babies, and mothers need, really stood out to me*.” For some students, this reality reinforced the responsibility of adaptability in nursing. One student explained this perfectly: “*working with limited resources forced me to focus on what really matters: the patient. I realized that nursing is more than following protocols; it’s about adapting, advocating, and prioritizing care where it counts* (Participant 8).”

Comparisons with Canadian healthcare systems further deepened these realizations. Students became acutely aware of the waste and overuse of supplies in Canada when contrasted with the efficiency and sustainability in Tanzania. One student’s narrative reveals the amount of healthcare system waste, stating, “We *throw out so much wound care and other supplies and never think about it. . . but seeing how one item can be so meaningful to others will always stay in my mind when I’m working in healthcare*.” One student reflected on the resource limitations leading to inequities in care delivery, highlighting “*how they used something like a glove for a tourniquet. It showed me how they provide the best care with so little*” (Participant 6). Another student observed, “*In Canada, we have so much that it leads to waste. In Tanzania, I saw people using plastic water bottles as tools to store a disinfectant. . . it was efficient and thoughtful. It made me reflect on how much more sustainable we could be*” (Participant 9).

I knew about waste before, but seeing it firsthand really changed me. In Canadian hospitals, you go to do a wound dressing and find 100 unused supplies that might be thrown out. If I wasted the same way in Tanzania, the impact would be so drastic. (Participant 1)

Students also witnessed how healthcare professionals need to acknowledge their privilege, both personally and at a systemic level. These discussions went beyond the level of individual care provision to consider larger impacts on care delivery and to think about equity-oriented practices. Participant 10 recalled how debriefing and reflection with the mentorship of a local healthcare staff helped prepare them to avoid a “*white saviour complex*” and create a safe experience for community members. The student described a hypertension screening clinic in the Maasai community, stating, “*At first, it felt like we were just telling people they had high blood pressure, but the local doctor explained it was about building trust and helping them feel comfortable. That’s not something I could have learned from a textbook*” (Participant 10). Ultimately, these experiences led to a mindset shift that extended beyond clinical skills into personal growth. One student eloquently described a lasting transformation: “*I’m a changed person, to see and witness it firsthand*” (Participant 5). Another student reflected on this transformational change, explaining, “*I went in thinking I could just ‘apply my skills,’ but I learned so much about listening and learning from local providers. It changed how I see my role as a nurse*” (Participant 10). These reflections highlight how learning in resource-limited settings reshaped students’ definitions of equity in healthcare in a cross-cultural learning experience.

## Discussion

This study explored undergraduate nursing students’ experiences of developing cultural competence during an IICP in Tanzania. Students’ experiences reflected Campinha-Bacote’s (2018) model of *cultural competemility*, which views cultural competence as interconnected, evolving processes. Students’ stories can be interpreted through Campinha-Bacote’s (2002) construct of cultural encounters, which appears to function as catalytic moments that initiate shifts in awareness, challenge prior knowledge structures, and stimulate reflexive learning. Rather than progressing linearly through competence domains, students’ narratives suggest recursive movement between encounters, reflection, and identity reconstruction.

Consistent with narrative inquiry’s emphasis on temporality, students’ accounts revealed learning as an evolving process rather than a discrete outcome, with participants frequently contrasting their initial expectations, in-country experiences, and retrospective interpretations following their return to Canada. Participants demonstrated growth across awareness, knowledge, skill, encounters, and desire, emphasizing humility as foundational to learning. Interactions with Tanzanian healthcare professionals and patients broadened their understanding of culturally appropriate care and underscored the value of respect, reciprocity, and relational engagement in global nursing practice. Students’ narratives illustrate how this IICP in Tanzania functioned not merely as a clinical site but as a meaning-shaping place that disrupted familiar assumptions about healthcare, professional roles, and resource availability. The unfamiliar clinical, social, and cultural environment shaped how students developed cultural competence as they made sense of relational and clinical encounters.

### Clinical Preparation and Supervision

Consistent with the existing literature, the findings highlight the importance of thorough preparation prior to international placements (Caldwell & Purtzer, 2014; Kokko, 2011). One notable gap identified was insufficient preparation related to local cultural practices and language training. Language barriers emerged as a significant challenge, limiting students’ ability to conduct assessments and build rapport with patients. Similar challenges have been documented in prior research, identifying language as one of the most significant obstacles during international clinical placements (Kelleher et al., 2016; Kumwenda et al., 2014; Unver et al., 2021). Clinical instructors or preceptors in comparable contexts have also expressed greater frustration with language barriers than cultural differences (Newton et al., 2018). Despite these difficulties, students employed creative communication strategies and interprofessional teamwork to bridge language barriers, thereby enhancing their empathy and cultural sensitivity. This concurs with our findings, in which students described faculty supervisors as essential to their motivation and learning and to making them feel safe during the IICP.

### Reflection and Cultural Self-Awareness

Exposure to unfamiliar cultural and clinical contexts prompted students to challenge assumptions, engage in critical reflection, and develop adaptability in complex environments. Students’ narratives positioned immersion experiences as moments of narrative tension, where previously held assumptions were disrupted. A unique feature of this placement was students’ engagement in *reflection-in-action*, involving real-time journaling and interprofessional debriefings rather than the traditional end-of-shift reflection model. This continuous reflection enabled them to integrate learning directly into practice, deepening cultural understanding and emotional resilience. Such findings support prior research suggesting that classroom instruction alone cannot fully achieve cultural learning in global contexts (Johnston et al., 2023).

Reflection and dialogue were also identified as key mechanisms for professional growth. Consistent with Ulvund et al. (2023), structured reflection and peer discussion helped students process emotional experiences, recognize biases, and consolidate learning. Reflection enabled them to explore their cultural identities and consider how personal beliefs and values shape clinical interactions. This echoes Curtis et al. (2025), who emphasize that health professionals should be aware of their cultural frameworks to deliver equitable and person-centered care that is consistent with advancing cultural humility and toward culturally safe care.

### Advancing Cultural Humility and Cultural Safety in Clinical Settings

Although cultural competence remains central to person-centered care and equity-oriented practice, recent literature and calls to action within nursing education increasingly emphasize the importance of cultural humility and cultural safety (Curtis et al., 2019; Curtis et al., 2025). From an intersectional perspective, students acknowledged that culturally responsive care cannot be reduced to static cultural attributes, as patients’ experiences are shaped by intersecting social identities and structural conditions (Lekas et al., 2020). Cultural humility foregrounds lifelong self-reflection, recognition of patients’ contextual expertise, attentiveness to power imbalances, and a sustained commitment to learning (Tervalon & Murray-García, 1998; Foronda et al., 2016). Rather than implying mastery, this orientation frames culturally responsive practice as an ongoing, relational process (Lekas et al., 2020).

Students’ narratives suggest that their learning extended beyond the acquisition of culturally specific knowledge and skills toward reflexive and relational ways of knowing consistent with cultural humility. Participants’ descriptions of “unlearning” and evolving self-awareness reflect processes of critical self-examination and openness to new perspectives. These experiences align with the literature that positions cultural humility as foundational to ethical, patient-centered nursing practice. Furthermore, recognition of cultural safety shifts attention from provider competencies to how care is experienced by those receiving it (Ramsden, 2002). Cultural safety requires critical awareness of power relations, systemic inequities, and the historical contexts that shape healthcare interactions (Curtis et al., 2019).

Participants’ reflections on privilege, positionality, and adaptation to locally grounded practices illustrate emerging awareness of these structural and relational dimensions of care. Cultural safety requires healthcare professionals and their organizations to influence healthcare to reduce bias and achieve equity within the workforce and work environment (Curtis et al., 2025). Our findings suggest that the IICP fostered not only cultural competence, as conceptualized by Campinha-Bacote (2002), but also the reflexive orientation characteristic of cultural humility and the broader relational and structural awareness central to cultural safety (Curtis et al., 2025).

### Strengths-Based and Decolonizing Perspectives

While previous studies often describe ICP through a deficit lens, focusing on limited resources or breaches in practice standards, this study revealed a distinct strengths-based perspective. Unlike the findings of Andreassen et al. (2025), which reported inadequate resources and infection control or hygiene violations, participants in this study emphasized the innovation, skill, and resilience of Tanzanian healthcare providers. Students described witnessing resourcefulness, creativity, and strong teamwork, reframing what might otherwise be perceived as constraints into examples of excellence within the local context. This perspective signifies a shift from deficit-oriented to a strengths-based approach to global learning and aligns with contemporary calls to decolonize global health education (CASN, 2025). Rather than focusing on what low-resource environments lack, students recognized and valued local expertise, ingenuity, and culturally grounded approaches to care. Importantly, these findings reflect a shift beyond cultural competence, often framed as the acquisition of knowledge or skills, toward the development of cultural safety, which emphasizes reflexivity, awareness of power dynamics, and healthcare providers’ responsibility for creating environments experienced as safe by those receiving care. As outlined in the CASN *Cultural Humility and Cultural Safety Framework* (2025), nursing education must intentionally support learners in critically examining their positionality, challenging colonial assumptions, and engaging in ongoing relational accountability. The strengths-based perspectives demonstrated by participants suggest that, when thoughtfully designed, IICP can serve as powerful pedagogical spaces to advance cultural safety rather than reinforce hierarchical or colonial narratives.

### Interprofessional Learning and Collaboration

The interprofessional design of the placement further enhanced students’ development of collaboration, communication, and leadership skills. Working alongside peers from other disciplines allowed participants to gain insight into different professional roles and the collective nature of healthcare delivery. Consistent with Costello et al. (2017) and Reeves et al. (2016), students reported improved understanding of teamwork, which created the context for developing cultural competence and for culturally responsive interprofessional collaboration. The integration of cultural learning within interprofessional contexts encouraged ethical reflection and critical dialogue across disciplines, suggesting that such placements provide a rich setting for developing both interprofessional competence and cultural awareness simultaneously.

### Understanding Equity Within a Global Health Context

Students also reported heightened awareness of health inequities and the social determinants of health. Immersion in resource-limited environments encouraged reflection on how multiple intersecting issues converge to shape access to healthcare. However, unlike previous studies that emphasize discomfort, pity, or helplessness in clinical settings, students in this study viewed these experiences as opportunities to appreciate local resilience (Caldwell & Purtzer, 2014). These insights parallel the findings of Johannessen et al. (2014), Murray (2015), and Gower et al. (2023), who noted that global experiences foster a deeper understanding of social justice and advocacy, thereby enabling students to adopt a strengths-based approach to international clinical placements.

## Implications for Practice and Education

The findings highlight the value of combining international and interprofessional experiences as high-impact educational strategies for developing cultural competence and leadership in nursing education. Immersive, collaborative learning experiences allow students to engage in authentic, patient-centered practice while reflecting on their personal and professional identities. Faculty support, structured reflection, and guided debriefing are essential for facilitating meaning-making and sustaining learning outcomes (Jeffreys, 2016; Kaihlanen et al., 2019).

Consistent with PAR principles, the study’s findings serve as a catalyst for ongoing curricular action rather than an endpoint of inquiry. The research team is actively using insights from students’ narratives to guide revisions to the IICP structure and processes. Two students are returning to Tanzania with the Teach Tanzania team and are actively working to incorporate additional cultural training to enhance the development of culturally safe practice. This will include enhanced pre-departure training with expanded cultural and contextual preparation, and more intentional scaffolding of interprofessional learning to ensure integration rather than siloed preparation. In collaboration with colleagues, particular attention is being directed toward strengthening language preparation, cultural education, engagement in pre-experience reflexive practice, and structured reflective pedagogies. Specific initiatives also include incorporating participant narratives into curriculum resources and simulation-based learning activities, enabling future students to engage with authentic experiential accounts of cultural humility and cross-cultural care. These developments aim to ensure that knowledge co-generated through the study informs tangible educational practices, thereby sustaining the participatory action cycle and informing subsequent IICPs. This study also adds to ongoing discussions about methodological rigour. There remains a need for high-quality studies investigating the effectiveness of nurses’ cultural competence interventions, particularly technology-based ones (Oikarainen et al., 2019; Osmancevic et al., 2025). While some interventions have demonstrated improvements in patient-related outcomes, overall evidence remains limited due to inconsistent designs and a lack of validated measurement instruments, a concern echoed by Osmancevic et al. (2025). Future research should prioritize mixed-methods designs and validated outcome measures to evaluate dimensions of cultural competence development.

Experiential learning opportunities, such as ICP, IICP placements, simulation, and service learning, should be intentionally embedded within nursing education. These approaches strengthen the affective and behavioural aspects of cultural competence and prepare students for practice in increasingly diverse and interconnected healthcare environments. Adopting a strengths-based, reciprocal learning framework also encourages students to recognize the expertise of global partners and to approach cultural learning with humility and mutual respect.

## Strengths and Limitations

This study provides new insight into how cultural competence develops at the intersection of international and interprofessional learning. Methodologically, students were intentionally included as co-authors; although they did not have formal training in research methodologies, their stories were prioritized and viewed as a strength for making sense of their IICP experiences. The participatory narrative inquiry design engaged students as co-researchers and enhanced reflexivity and credibility. However, the small, all-female sample and single-site context may limit the transferability of the findings. Our all-female team may have shaped rapport, disclosure, and the meanings foregrounded in narratives. Students’ social, cultural, and educational positions informed how experiences were narrated and interpreted, whereas faculty perspectives shaped the analytic framing and theoretical integration. Given methodological variability in the broader evidence base, future research should use rigorous mixed-methods designs and longitudinal follow-up to assess learner, patient, and system outcomes. Future studies should explore diverse cultural and institutional contexts and assess the long-term influence of IICP on nursing practice and patient outcomes.

## Conclusion

IICP emerged as a high-impact, ethically grounded learning experiences that advance undergraduate nursing students’ cultural competence. Immersion alongside interprofessional teamwork and structured reflection in action fostered self-awareness, empathy, cultural competence, and confidence, while also revealing practical needs for stronger language preparation and clear, supportive supervision. Notably, students adopted a strengths-based lens, recognizing the innovation and resilience of local providers, rather than deficit-focused interpretations of low-resource contexts, aligning with decolonizing approaches to global nursing education. These findings support the intentional embedding of IICP, within nursing curricula to cultivate culturally safe, equity-oriented practice. Cultural competence is lifelong; well-designed IICP helps bridge classroom knowledge and clinical reality, preparing graduates for culturally competent care.

## Supplemental Material

sj-docx-1-gqn-10.1177_23333936261450418 – Supplemental material for Becoming Culturally Competent: Transformative Learning in an International, Interprofessional Nursing Placement Supplemental material, sj-docx-1-gqn-10.1177_23333936261450418 for Becoming Culturally Competent: Transformative Learning in an International, Interprofessional Nursing Placement by Rachel A. Elliott, Heather Sweet, Natalie Bownes, Bayan Nasra, Kelsey Schumacher, Kelly-Anne Vanderdoe, Gabriela Digesu, Eunice Kungu, Grace Okello, Emma Crew, Mia Chauvin and Kendall Soucie in Global Qualitative Nursing Research
