# Supplementary material for: Becoming Culturally Competent: Transformative Learning in an International, Interprofessional Nursing Placement
Source: Glob Qual Nurs Res. 2026 May 15;13:23333936261450418. doi: 10.1177/23333936261450418 (PMC13180154; doi:10.1177/23333936261450418)
Supplement: sj-docx-1-gqn-10.1177_23333936261450418 – Supplemental material for Becoming Culturally Competent: Transformative Learning in an International, Interprofessional Nursing Placement [file sj-docx-1-gqn-10.1177_23333936261450418.docx]

**Supplemental Material**

**Co-Created Semi-Structured Interview Guide**

- 1. Before you travelled to Tanzania, what were your expectations regarding this unique clinical consolidation experience in Tanzania? Can you tell me a story or moment that captures what you were anticipating at that time?
  2. What was the best part of this clinical consolidation experience? Can you give me an example…
  3. Can you tell me about a time when you felt like you learned something that you wouldn’t have been able to learn otherwise in the BScN program? Can you tell me about a moment during the placement that stands out as particularly meaningful or impactful for you? What was happening, and why does this moment stay with you?
  4. What could be improved to make this experience more effective to meet your learning needs?
  5. Did you feel that this experience helped to improve your ability to provide culturally safe care? Why or why not? If not, what could have helped you to do this more effectively?
  6. What training or education could have been provided to help you prior to this clinical experience?
  7. What barriers, if any, did you face in being able to access or participate in this international placement? Can you describe what was happening and how you navigated those barriers?
  8. Did you feel that collaboration with the teachers in training from the Faculty of Education was beneficial to your learning? How did that interaction influence your learning or perspective?
  9. What are some of the strengths of offering international clinical experiences to nursing students? What are some of the benefits to offering interprofessional clinical experiences to nursing students?
  10. What are some suggestions you may have of things that could be added to sustain this experience for future students?
  11. Do you have any concerns related to this clinical experience? If so, what specifically and how could they be avoided or prevented in the future?
  12. Looking back now, how do you think this experience shaped the way you see yourself as a nursing student or future nurse? Can you share an example that illustrates this?
  13. Would you like to see this type of clinical consolidation experience continue to be offered in this program? Why or why not? If a future nursing student were preparing for this experience, what stories or lessons from your own journey would you want to share with them?
  14. Is there a story, moment, or reflection that we haven’t talked about yet that feels important for understanding your experience with the Teach Tanzania clinical placement?

**Additional probing using the DICE approach**

To ensure rigour in qualitative research, additional probing questions using the DICE approach (Robinson, 2023) will be used in qualitative interviews.

**Descriptive Detail Probes**

- Could you give me some examples?
- You just told me about…. I’d also like to know about….
- Tell me more about what happened when … was going on…”
- Could you tell me more about your thinking on that?

**Idiographic Memory Probes**

- What stands out in your mind about that?
- “Can you tell me an example of when that happened?”
- You mentioned…. Could you tell me more about that?
- You mentioned ….could you describe a specific example of when that happened?

**Clarifying Probes**

- What do you mean by ….?
- You mentioned …. can you tell me what that means to you?
- What do you mean by….”
- Can you tell me more about that?
- I’m not quite sure I understood …could you clarify what you mean by….
- This is what I thought I heard…Did I understand you correctly?
  - - So what I hear you saying is…”
    - What do you mean by ….”

**Explanatory Probes**

- Why do you think that this happened?
- Could you please tell me more about…
- Could you give me some examples?
- What stands out in your mind about that?
- Can you give me an example of…
- Why do you think that happened?
- You just told me about…. I’d also like to know about….

*Note.* Adapted from Robinson, O. C. (2023) Probing in qualitative research interviews: Theory and practice. *Qualitative Research in Psychology, 20*(3), 382-397. <https://doi.org/10.1080/14780887.2023.2238625>
